# Nurses’ Perspectives on Leadership Styles and Work Engagement in Jordan Hospitals: A Cross‐Sectional Study

**DOI:** 10.1155/jonm/5508128

**Published:** 2026-08-26

**Authors:** Amal Alkhatib, Zaid Al-Hamdan

**Affiliations:** ^1^ Faculty of Nursing, Jordan University of Science and Technology, Ar-Ramtha, Jordan, just.edu.jo; ^2^ The Faculty of Graduate Studies, Jordan University of Science and Technology, Ar-Ramtha, Irbid, Jordan, just.edu.jo

**Keywords:** Jordan, leadership, leadership style, nurse engagement, nursing, work engagement

## Abstract

**Aim:**

The study examined the relationship between leadership styles and work engagement among nurses from their perspectives.

**Background:**

Nurses frequently face substantial stress, burnout, and emotional weariness as a result of high patient care demands and staffing shortages. Keeping nurses engaged is crucial for healthcare firms looking to retain employees while offering high‐quality care.

**Methods:**

A cross‐sectional design was adopted, using a convenience sampling technique, and descriptive and correlational analyses were carried out. A total of 337 registered nurses were recruited. Data were collected from participants using self‐administered questionnaires, including the Utrecht Work Engagement Scale (UWES), which was used to assess nurses’ work engagement.

**Results:**

Directive leadership is perceived as the dominant leadership style by registered nurses in Jordanian hospitals. When examining the dominant leadership style as perceived by registered nurses, the directive leadership style achieved the highest mean score (*M* = 23.55, SD = 5.67), followed by the achievement‐oriented style (*M* = 22.09, SD = 4.93). In third place, the participative leadership style was accounted for (*M = 21.85, SD = 6.45*). The supportive leadership style ranked last with a mean score of 21.55 (SD = 5.81).

**Conclusion:**

Effective leadership styles are associated with higher levels of work engagement among nurses.

**Implications for Nursing Management:**

These findings could guide policymakers in establishing organizational policies to train nurse managers in effective leadership styles that enhance nurses’ engagement and quality of life, while also enriching the factors that may enhance nurses’ motivation and reduce their turnover intention. This study is descriptive and has an IRB number 34/165/2023.

## 1. Introduction

### 1.1. Background

Effective leadership is widely recognized as a critical determinant of organizational success and a key factor influencing healthcare outcomes [1, 2]. Leadership plays an essential role in managing human resources and enhancing organizational performance; however, its effectiveness varies with the context and the leadership approach adopted. Despite extensive theoretical perspectives on leadership, including behavioral, relational, and trait approaches [3], there remains a lack of consensus on which leadership styles are most effective at improving nurses’ work engagement.

In nursing settings, effective leadership is particularly important for enhancing nurse performance, ensuring patient safety, and maintaining high‐quality care. However, nurses continue to experience high levels of stress, burnout, and workforce shortages globally, which are negatively associated with their engagement at work [4]. This highlights the need to better understand how leadership styles can mitigate these challenges and promote work engagement.

According to path–goal leadership theory, leadership styles such as participatory, supportive, directive, and achievement‐oriented leadership influence employees’ motivation and performance. While participatory and supportive leadership styles are often associated with improved staff well‐being and engagement, directive leadership may be more effective in structured clinical environments [5]. Nevertheless, empirical evidence regarding the relationship between these leadership styles and nurses’ work engagement remains inconsistent across different healthcare contexts.

Furthermore, although numerous studies have been conducted in Western countries, little research has examined this relationship in Middle Eastern contexts, particularly in Jordan. Given the unique characteristics of the Jordanian healthcare system, including its organizational structure and workforce distribution, it is important to investigate how leadership styles influence nurses’ work engagement in this setting.

### 1.2. Statement of the Problem

Nurses play a vital role in improving patient outcomes; however, poor working conditions, limited career opportunities, and inadequate compensation contribute to high turnover rates and workforce shortages, particularly in public hospitals. These challenges not only affect healthcare systems but also negatively influence nurses’ work engagement and job satisfaction.

Globally, nurse shortages remain a pressing concern, and high turnover rates further exacerbate this issue [6]. In Jordan, staffing shortages are significantly affecting the quality of care and increase burnout among nurses [7]. Although previous studies have demonstrated that higher work engagement is associated with reduced turnover intentions and improved patient outcomes [8], the factors that enhance nurses’ engagement remain insufficiently understood in this context.

Despite the recognized importance of leadership in influencing nurse outcomes, limited research has specifically examined the relationship between leadership styles and work engagement among nurses in Jordan. Therefore, this study aims to address this gap by investigating nurses’ perceptions of leadership styles and their relationship with work engagement.

### 1.3. Significance of the Study

This study contributes to the literature by examining the relationship between leadership styles and nurse work engagement in Jordan. The findings have practical significance for nursing management and healthcare policy, as they inform the development of strategies to improve nurse retention, reduce burnout, and enhance patient care outcomes. Furthermore, this study provides a foundation for future research on leadership in hospital settings.

### 1.4. Aims and Objectives of the Study

This study aims to examine the relationship between leadership styles and work engagement among nurses from their perspectives.

The objectives of the study are as follows:

• To find out what leadership style is dominant at MOH hospitals (Al‐Basheer and Prince Hamza) in Jordan, as perceived by staff nurses.
• To identify the level of work engagement of the nurse.
• To find the relationship between leadership styles and the work engagement of nurses.
• To find the relationship between work engagement and demographic variables (age, gender, years of experience, marital status, department, and hospital).

### 1.5. Theoretical Framework

Many health theories and models can be used to assess leadership style and levels of work engagement. In this study, we proposed using path–goal theory to assess the leadership style adopted by managers and as perceived by staff. Work engagement was used as a construct to assess staff well‐being and work engagement. There are various approaches used in the field of leadership, such as trait and personality, behavioral, contingency or situational, transactional, transformational, and self‐leadership. Path–goal theory falls under the contingency approach, which focuses on the interaction between variables in a leadership scenario and patterns of leadership behavior. On the other hand, these studies offer another common way of studying leadership. Contingency or situational theories are based on the assumption that no single leadership pattern applies to all situations. The key leadership contingency models are the leadership effectiveness model, the decision‐making model, path–goal theory, and the situational leadership theory of Hersey and Blanchard [9].

**FIGURE 1:**
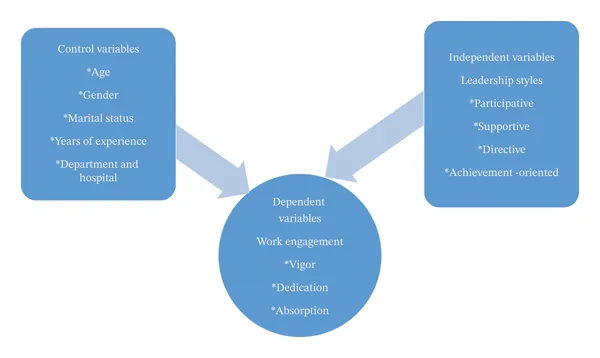
Conceptual framework showing the relationships between leadership styles and nurses’ work engagement.

The Utrecht Work Engagement Scale (UWES) defines work engagement as a favorable mindset toward one’s employment. Work engagement consists of three dimensions: energy, dedication, and absorption. Based on the reviewed literature, this research study conceptualizes the following relationships among variables (Figure 1).

Independent variables (leadership styles) represent job resources, while work engagement (vigor, dedication, and absorption) represents the study outcome.

Demographic variables were included as control variables.

#### 1.5.1. Path–Goal Leadership Model

Path–goal theory research focuses on investigating the relationships between leadership behaviors and satisfaction, as well as the effects of other moderator variables such as task characteristics. For example, evidence suggests that situational variables may influence directive and supportive leadership behaviors and their effects on outcomes such as subordinates’ and job satisfaction [11].

#### 1.5.2. Job Demands–Resources (JD‐R) Model of Work Engagement

Work engagement is a unique aspect of nursing that guides nurses and supervisors as they develop their profession in an environment that promotes safe and effective care [12]. In everyday life, “engagement” encompasses involvement, dedication, passion, excitement, absorption, focused effort, and energy. For example, the Merriam‐Webster dictionary characterizes participation as “emotional,” “participation or commitment,” and “the state of being in gear.” Demerouti et al. established the JD‐R model in 2001, which remains the most widely utilized theoretical framework today in research about work engagement [13, 14].

The JD‐R model of engagement is a helpful tool for promoting involvement, with clear implications for practice [15]; the authors in [16] concluded that three instruments were used to assess registered nurses’ work engagement in 17 quantitative studies, using a version of the UWES developed in [17]. According to Cole et al., the UWES results are similar to those of the Maslach Burnout Inventory (MBI), rendering it redundant as a measurement instrument. Some have criticized the technique utilized to establish the UWES. The majority of questions within the UWES also appear. These items may be similar to, or even identical to, other well‐known measures of employee well‐being, such as job satisfaction, positive affect, and organizational commitment. Rich et al., drawing on Kahn’s study and the definition of engagement, highlight the questions used in the UWES that confound work engagement antecedents and work involvement itself [18].

#### 1.5.3. Integration Between Models

Although path–goal theory and the JD‐R model have been frequently used independently, combining them provides a more complete picture of nurses’ work engagement. Leadership styles defined in path–goal theory, such as supportive, participative, directive, and achievement‐oriented, can be viewed as valuable job resources within the JD‐R paradigm.

These leadership styles may help minimize workplace demands, such as stress, workload, and emotional strain. While job demands may reduce work engagement, adequate job resources, including effective leadership behaviors, can foster vigor, dedication, and absorption. This study adopts an integrated approach to examine the relationship between path–goal leadership styles and nurses’ work engagement, as well as increasing job resources, such as support, motivation, and independence. As a result, strong leadership can help nurses achieve higher levels of work engagement.

## 2. Methodology

### 2.1. Study Design

A quantitative, cross‐sectional, descriptive correlational design was used to examine the relationship between leadership style and nurses’ work engagement.

### 2.2. Setting

This study was conducted in governmental and semigovernmental hospitals located in the central region of Jordan, specifically at Al‐Basheer and Prince Hamza hospitals. These hospitals were selected due to their high patient volumes, diverse specialties, and accessibility for ethical approval procedures.

### 2.3. Sampling Technique and Sample Size Calculation

A total of 337 registered nurses participated in the study. Convenience sampling was used due to accessibility and feasibility considerations; however, this approach may limit the generalizability of the findings. The sample size was calculated based on the total number of registered nurses at the selected hospitals, according to the 2023 ministry of Health report (Al‐Basheer Hospital: 1417; Prince Hamza Hospital: 546). Using the Raosoft sample size calculator, a minimum sample of 322 participants was required at a 95% confidence level and a 0.05 margin of error; however, 337 nurses were recruited to ensure sufficient statistical power.

### 2.4. Instruments

This study employed two instruments. The UWES, which includes a validated Arabic translation, was used to assess nurses’ work engagement. The scale had previously been validated in Arabic‐speaking people; no extra translation procedures were necessary.

The leadership styles questionnaire was translated into Arabic using a forward–backward translation method. Initially, a bilingual specialist translated the instrument from English to Arabic. To ensure accuracy, a second independent translator translated the Arabic version back into English.

To ensure cultural appropriateness, the translated version was reviewed by a panel of experts in nursing and healthcare management. Minor modifications were made to improve clarity and relevance to the Jordanian context. The final version was pilot‐tested with a small group of nurses to ensure clarity and comprehensibility. Data were collected using a self‐administered questionnaire consisting of three parts.

#### 2.4.1. The Sociodemographic Data

Part 1 included seven demographic questions: age, gender, marital status, job title, years of experience, unit of work, and hospital of work. This information was used to explore the relationships between the main study variables and demographic data.

#### 2.4.2. Path–Goal Leadership Questionnaire (PGLQ)

Part 2 assessed leadership styles as perceived by survey respondents and compared them with those perceived by staff. The PGLQ consists of 20 items measured on a 7‐point Likert scale, providing respondents with 7 options to express their level of agreement (*never* = 0 to *always* = 7). The tool measures directive, supportive, participative, and achievement‐oriented leadership styles from the subordinate perspective [19, 20].

To ensure data interpretation aligned with the instrument’s scoring guidelines, four items (5, 8, 13, and 19) with negative wording underwent reverse scoring. Responses on the 7‐point Likert scale were adjusted: a score of 1 became 7, 2 became 6, 3 became 5, 4 remained 4, 5 became 3, 6 became 2, and 7 became 1. This reverse scoring process standardized the measurement, so that elevated scores consistently represented a greater degree of the associated leadership style before the calculation of subscale scores.

Cronbach’s alpha for the subscales ranged from 0.73 to 0.79 in previous studies, indicating acceptable reliability.

#### 2.4.3. UWES

Part 3 assessed nurses’ work engagement using the UWES [17], which includes 17 items divided into three subscales: Vigor VI (6 items), Dedication DE (5 items), and Absorption AB (6 items). All items are scored on a 7‐point frequency scale (0 = *never* to 6 = *always*). The scale demonstrates strong psychometric properties, with Cronbach’s alpha ranging from 0.80 to 0.90.

In this study, internal consistency reliability was assessed using Cronbach’s alpha coefficients, which showed high reliability for both tools: PGLQ (*α* = 0.8) and UWES (*α* = 0.78). These values indicate strong internal consistency for the instruments used in the Jordanian nursing context.

### 2.5. Ethical Considerations and Human Rights

The Institutional Review Board (IRB) at Jordan University of Science and Technology provided ethical approval for this work. Participation in the study was voluntary, and all participants provided informed consent electronically before data collection. Participants were guaranteed secrecy and anonymity, and no personally identifiable information was collected.

They were also advised that they might withdraw from the study at any time without penalty. All data were securely saved and utilized only for research purposes.

### 2.6. Data Collection Procedure

The researcher conducted data collection between January and March 2024. Nurses were invited to participate through official Facebook groups, WhatsApp, and Messenger, with coordination by head nurses. Online data collection was used to ensure a broader geographical reach and to collect data effectively from nurses across various healthcare settings.

The online survey included a consent page, and participants could proceed only after confirming their consent. The survey link was disabled once 337 responses were received. Personal identifiers were not collected to ensure anonymity.

### 2.7. Statistical Analysis

The collected data were exported into SPSS software for analysis. Before analysis, a preliminary data scan was conducted to identify missing values and outliers, and normality was assessed. Pearson correlation and multivariate analysis were conducted after confirming assumptions of linearity, multivariate normality, homogeneity of variance–covariance matrices (Box’s M test), and absence of multicollinearity. The Pearson correlation coefficient was computed to examine the relationship between variables, and 95% confidence intervals were reported where applicable. Reverse‐scored items in the PGLQ were recorded before calculating subscale scores and the total mean value.

## 3. Results

### 3.1. The Demographic Characteristics of the Participants

A total of 337 registered nurses participated in the study. The majority were female nurses (74.5%), and more than half of the sample were married (59.6%). Participants were recruited from Prince Hamza Hospital (49.0%) and Al‐Basheer Hospital (51.0%), with nearly equal representation from the internal medicine, surgery, and emergency departments. The mean age of participants was 30.81 years (SD = 6.20), and the mean work experience was 6.01 years (SD = 5.67).

Nurses’ sociodemographic characteristics *N* = 337.

Gender: male = 86 (25.5), female = 251 (74.5).

Marital status: single = 136 (40.4), married = 201 (59.6).

Hospital: Prince Hamza = 165 (49), Al‐Basheer = 172 (51).

Department: internal medicine = 112 (33.2), surgical = 110 (32.6), emergency = 115 (34.1).

Age/years: mean (30.81), SD (6.20).

Experience: mean (6.01), SD (5.67).

### 3.2. Dominant Leadership Style of Nurse Leaders

Directive leadership is perceived as the dominant leadership style by registered nurses in Jordanian hospitals.

When examining the dominant leadership style as perceived by registered nurses, the directive leadership style achieved the highest mean score (*M* = 23.55, SD = 5.67), followed by the achievement‐oriented style (*M* = 22.09, SD = 4.93). In third place, the participative leadership style was accounted for (*M = 21.85, SD = 6.45*). The supportive leadership style ranked last with a mean score of 21.55 (SD = 5.81).

### 3.3. What is the Level of Work Engagement Among Nurses at Jordanian Public Hospitals?

When comparing the work engagement subscales, the highest mean score was found for dedication (*M* = 3.86, SD = 1.43), followed by vigor (*M* = 3.45, SD = 1.36) and absorption (*M* = 3.43, SD = 1.36). However, when the three subscales were categorized into three levels based on the interquartiles, the results presented in Table 1 showed that approximately 46.0% of the sample reported a moderate level of work engagement across all three subscales.

**TABLE 1:** Ranking of work engagement subscales among nurses at Jordanian public hospitals.

Work engagement	Mean out of 6	SD	^∗^Low *n* (%)	^∗^Moderate *n* (%)	^∗^High *n* (%)
Dedication	3.86	1.43	86 (25.5)	160 (47.5)	91 (27.0)
Vigor	3.45	1.36	90 (26.7)	152 (45.1)	95 (28.2)
Absorption	3.43	1.36	88 (26.1)	157 (46.6)	92 (27.3)

^∗^Categorized based on intraquartiles.

### 3.4. What is the Relationship Between Leadership Styles and Nurses’ Work Engagement?

Pearson’s product‐moment correlation demonstrated that the leadership styles have a significant positive correlation with all work engagement subscales. Directive leadership style has a moderately significant correlation with all work engagement subscales, with absorption (*r* = 0.513) having the highest correlation, followed by vigor (*r* = 0.499) and dedication (*r* = 0.498). Moreover, the supportive leadership style exhibits the strongest correlation with vigor (*r* = 0.526), followed by absorption (*r* = 0.486) and dedication (*r* = 0.473). Participative leadership style also shows a moderately significant correlation with vigor (*r* = 0.496), absorption (*r* = 0.491), and dedication (*r* = 0.483). Finally, achievement orientation showed a weak but significant relationship with all work engagement subscales. However, the highest correlation was with absorption (*r* = 0.390), followed by dedication (*r* = 0.357) and vigor (*r* = 0.353). Moreover, all leadership styles demonstrate a statistically positive relationship with total nurses’ work engagement scores. The highest correlation was noted with the directive style (*r* = 0.525), while the lowest was with the achievement‐oriented style (*r* = 0.383) (Table 2).

**TABLE 2:** Relationship between leadership styles and nurses’ work engagement.

Leadership style	Work engagement			Total work engagement
	Vigor subscale	Dedication subscale	Absorption subscale	
Directive	0.499**^∗∗^**	0.498**^∗∗^**	0.513**^∗∗^**	0.525^∗∗^
Supportive	0.526**^∗∗^**	0.473**^∗∗^**	0.486**^∗∗^**	0.518^∗∗^
Participative	0.496**^∗∗^**	0.483**^∗∗^**	0.491**^∗∗^**	0.512^∗∗^
Achievement‐oriented	0.353**^∗∗^**	0.357**^∗∗^**	0.390**^∗∗^**	0.383^∗∗^

*Note:* The bold values in Table 2 indicate statistically significant correlations at *p* < 0.001.

^∗∗^Significant at *p* value < 001, *r* = 0–0.19 is regarded as very weak, 0.2–0.39 as weak, 0.40–0.59 as moderate, 0.6–0.79 as strong, and 0.8–1 as a very strong correlation.

### 3.5. What is the Relationship Between Nurses’ Work Engagement and Demographic Variables (Gender, Marital Status, Hospital, Department, Age, and Years of Experience)?

A multivariate analysis of variance (MANOVA) and Pearson correlation were used as inferential parametric statistical tests to explore the relationship between nurses’ work engagement subscales and demographic variables. The results in Table 3 revealed that nurses’ age was statistically significantly associated with the work engagement subscales, but the relationships were weak. However, workplace experience demonstrated a significant correlation only with the vigor and absorption subscales. Regarding the relationship with the overall work engagement score, both nurses’ age and experience show a significant association.

**TABLE 3:** Relationship between nurses’ work engagement and demographic variables (gender, marital status, hospital, department, age, and years of experience).

Variables	Overall MANOVA Wilks’ lambda (WL)	Category	Work engagement						Overall work engagement	
			Vigor		Dedication		Absorption			
			Mean	SD	Mean	SD	Mean	SD	Mean	SD
Gender	WL = 0.999	Male	3.53	1.37	3.73	1.44	3.43	1.25	3.55	1.30
	*F* = 0.119	Female	3.43	1.36	3.91	1.43	3.43	1.40	3.57	1.33
	*p* = 0.0.88	*F* value (*p* value)	0.311 (0.578)		1.019 (0.313)		0.00 (0.988)		0.013 (0.910)	

Marital status	WL = 0.999	Single	3.47	1.28	3.84	1.39	3.42	1.29	3.56	1.29
	*F* = 0.157	Married	3.45	1.42	3.87	1.46	3.44	1.42	3.44	1.41
	*p* = 0.925	*F* value (*p* value)	0.023 (0.880)		0.033 (0.855)		0.011 (0.917)		0.002 (0.967)	

Hospital	WL = 0.967	Prince Hamza	3.43	1.41	3.90	1.44	3.36	1.39	3.54	1.56
	*F* = 1.761	Al‐Basheer	3.48	1.31	3.82	1.42	3.50	1.34	3.59	1.30
	*p* = 0.152	*F* value (*p* value)	0.120 (0.730)		0.231 (0.631)		0.840 (0.360)		0.093 (0.760)	

Department	WL = 0.975	Internal Medicine	3.44	1.42	3.71	1.41	3.35	1.42	3.49	1.36
	*F* = 1.398	Surgery	3.44	1.35	3.85	1.49	3.43	1.42	3.56	1.36
	*p* = 0.213	Emergency	3.48	1.33	4.02	1.38	3.51	1.27	3.65	1.25
		*F* value (*p* value)	0.038 (0.963)		1.339 (0.263)		0.397 (0.673)			

Age/years		*R* value (*p* value)	0.180 (0.001)		0.158 (0.004)		0.171 (0.002)		0.178	0.001

Experience/year		*R* value (*p* value)	0.131 (0.016)		0.094 (0.086)		0.128 (0.019)		0.124	0.023

*Note:* R: Pearson correlation.

On the other hand, neither nurses’ gender, marital status, hospital type, nor department had a significant relationship with the vigor, dedication, and absorption subscales and the total nurse work engagement score.

## 4. Discussion

The results revealed that directive leadership was the most prevalent style, followed by achievement‐oriented, participative, and supportive styles.

### 4.1. Interpretation of Leadership Findings

The frequency of directive leadership seen in this study can be viewed from both theoretical and contextual perspectives. According to path–goal theory, directive leadership entails providing clarity, structure, and guidance, particularly in complex or high‐stakes settings such as healthcare. Higher power‐distance cultures are common in Middle Eastern healthcare organizations. In such situations, hierarchical systems are often tolerated, and employees may favor clear instructions and centralized decision‐making. Jordan’s public hospitals usually operate within structured, centralized systems, which may indicate greater reliance on directed leadership to maintain adherence to clinical procedures and patient safety. However, this result should be interpreted with caution because contextual factors such as workload, organizational circumstances, and individual characteristics were not adequately controlled for in the study.

This finding may also be attributed to cultural influences. As in other Middle Eastern countries, hierarchical organizational structures may be associated with nurses’ expectations for unambiguous leadership guidance. This may explain the apparent preponderance of directive leadership. Nonetheless, these findings represent associations rather than causal links due to the study’s cross‐sectional nature.

Furthermore, the vital nature of patient care necessitates rigorous adherence to clinical guidelines, which may be linked to the perceived usefulness of directive leadership in supporting task completion and reducing potential errors.

### 4.2. Finding in Relation to the JD‐R Model

The JD‐R model posits that the interaction of job resources and demands influences employee engagement. In the current study, leadership styles were viewed as a significant workplace resource that might increase nurses’ work engagement. However, essential job demands, such as workload, personnel shortages, emotional strain, and burnout, were not clearly evaluated. Future studies should include these elements to enable a more comprehensive use and evaluation of the framework.

### 4.3. Comparison With Previous Studies

The findings of this study align with previous research conducted in Rwanda, where directive leadership was also reported as the dominant leadership style [21]. This similarity may reflect the structured nature of healthcare environments in developing countries, where clear guidance and adherence are essential.

Conversely, studies conducted in Saudi Arabia by Al‐Yami et al. [22] and Ghana [23] reported a greater prevalence of transformational and participative leadership styles. These differences may be attributed to variations in organizational structures, leadership development practices, and cultural expectations across healthcare systems.

Overall, these findings suggest that leadership styles are context‐dependent and influenced by multiple factors, including organizational structures, cultural norms, and healthcare system demands. However, these comparisons should be interpreted with caution, as differences in study design and sample characteristics may also contribute to the observed variations.

### 4.4. Leadership and Work Engagement Relationship

The study also examined work engagement among nurses using the UWES. Dedication scored highest, followed by vigor and absorption, with nearly half of the participants reporting moderate levels of engagement. Supportive leadership showed the strongest correlation with engagement subscales, particularly vigor, suggesting that nurses feel more energized and committed when leaders are approachable, emotionally supportive, and attentive to their needs. According to the JD‐R model, supportive leadership can be considered an important job resource that helps employees cope with workplace demands and promotes work engagement. Therefore, supportive leadership may contribute particularly to the vigor component by replenishing nurses’ psychological resources and reducing the negative effects of job stress. Participative leadership was also linked to higher levels of work engagement, suggesting the importance of decision‐making and collaboration. Directive leadership showed moderate positive correlations with all work engagement subscales, suggesting that clarity and structure may promote participation but may not fully meet emotional or motivational needs. Achievement‐oriented leadership showed smaller relationships, showing that focusing solely on performance goals may not be enough to maintain engagement.

These findings point to the potential value of a balanced, situational leadership approach that incorporates directive, supporting, and participative behaviors in healthcare settings. However, these findings should be regarded with caution because the cross‐sectional design limits causal inference, and other environmental or organizational factors may have influenced the observed associations.

### 4.5. Potential Confounding Variables

Several factors may have influenced these results. The type of hospital, workload intensity, and nurses’ educational backgrounds can influence both perceptions of leadership and engagement levels. For example, nurses in high‐demand units may perceive directive leadership differently from those in less‐pressured settings. Similarly, differences in training or experience could affect how nurses respond to participative or supportive leadership. These variables were not controlled in the current study and should be considered in interpreting the findings.

### 4.6. Implications for Practice

The results have clear practical implications:

• Nurse leaders should adopt flexible, situational leadership strategies, combining directive guidance with supportive and participative approaches based on the clinical context.
• Training programs for nurse managers should emphasize emotional intelligence, team involvement, and adaptive leadership, enhancing engagement, retention, and patient care outcomes.
• Healthcare organizations should consider cultural and organizational contexts when developing leadership policies, ensuring that leadership styles align with both employee expectations and patient safety requirements.

### 4.7. Demographic Influences

Age was weakly and positively associated with work engagement, suggesting that more experienced nurses may possess better coping strategies and resilience. Workplace experience showed a weak correlation with vigor and absorption, indicating some benefit of familiarity with job demands. Gender and marital status were not significantly associated with engagement, supporting the notion that work engagement is primarily influenced by organizational and leadership factors rather than personal demographics.

### 4.8. Limitations

This study has several limitations. First, the cross‐sectional design prevents causal inference. Second, the study sample was limited to public hospitals, using convenience sampling, which may restrict generalizability. Third, potential confounding variables, such as workload, hospital type, and educational background, were not controlled. Future research should explore these factors and consider longitudinal designs to examine changes in leadership and engagement over time.

### 4.9. Conclusion

According to the data, all leadership styles were moderately positively associated with nurses’ work engagement. Among these, directive leadership showed the strongest relationship, followed closely by supportive and participative leadership styles, while achievement‐oriented leadership showed the weakest relationship.

## 5. Limitations

This study has some limitations that must be considered when evaluating the findings. First, utilizing a cross‐sectional design limits the ability to establish causal relationships. Second, the use of convenience sampling and the small geographic scope may limit the generalizability of the results. Third, data were acquired via self‐reported questionnaires, which could lead to response bias. Furthermore, key confounding variables, including workload, nurse‐to‐patient ratio, compensation levels, staffing sufficiency, organizational support, and individual characteristics, were not completely controlled, potentially influencing the results. Future research should adjust for these characteristics to better understand the specific impact of leadership styles on nurses’ work engagement. Furthermore, future research could benefit from adopting multivariable analytical tools, such as multiple regression analysis, to evaluate the relative contribution of different leadership styles to nurses’ work engagement while controlling for potential confounding factors. Finally, the data are time‐bound; therefore, they only provide a snapshot and may not reflect changes in work involvement.

## 6. Conclusions

This study looked into the relationship between leadership styles and nurses’ work engagement in healthcare settings. According to the data, all leadership styles had a moderately good relationship with nurses’ work engagement. Directive leadership had the strongest correlation, followed by supportive and participative leadership styles, while achievement‐oriented leadership had a weak correlation.

These findings highlight the potential value of a flexible and context‐sensitive leadership strategy in healthcare settings, incorporating both task‐oriented and supportive behaviors. Such an approach may help to create work settings that promote nurses’ engagement and well‐being.

However, the findings should be interpreted with caution due to the cross‐sectional design and the potential influence of unmeasured contextual factors. Further research, particularly longitudinal studies, is recommended to better understand these relationships.

## 7. Implications for Nursing Management

The findings of this study suggest that different leadership styles are associated with nurses’ work engagement, with directive leadership showing the highest relationship, followed by supportive and participative styles.

Healthcare organizations may consider adopting a balanced leadership approach that combines clear structure and guidance with supportive and participative practices. Practical strategies may include training nurse managers to provide clear expectations and direction while also fostering open communication, emotional support, and staff involvement in decision‐making.

Additionally, leadership development programs, mentorship initiatives, and the integration of leadership assessment into organizational policies may support more effective leadership practices and improve nurses’ engagement.

## Funding

No funding was received for this manuscript.

## Ethics Statement

Ethical approvals were obtained from the following institutions: Institutional Review Board (IRB) of Jordan University of Science and Technology; IRB of the Jordanian MOH; and IRB of Prince Hamza Hospital.

## Conflicts of Interest

The authors declare no conflicts of interest.

## Data Availability

The data that support the findings of this study are available on request from the corresponding author. The data are not publicly available due to privacy or ethical restrictions.
